# MULTISTAGE SAMPLING FOR TRANSLATIONAL COMMUNITY RESEARCH: ZOOMING OUT TO HONE IN ON PLACE-BASED HEALTH DISPARITIES

**DOI:** 10.1093/geroni/igac059.1451

**Published:** 2022-12-20

**Authors:** Daniel Rong Yao Gan

**Affiliations:** Simon Fraser University, Vancouver, British Columbia, Canada

## Abstract

​​Whereas researchers strive for generalizability, community-engaged research (CEnR) typically involves only a few specific communities. Drawing on Weberian ideal type, I outline the use of an innovative blended-methods approach to sample the communities in which CEnR practitioners would collect in-depth data. To complement typical practices of entering a community without preconceived ideas, understanding how communities in the sampling frame relate to one another is important for equigenic (place-based health equity) implementations. The selection of neighborhood communities from quadrants in 2x2 matrices allows pertinent concepts to emerge and relevant solutions to be drawn from thriving communities to aid program co-creation and implementation in other communities. For example, this has led to the identification of communities in British Columbia with differing socioeconomic status, social capital, and coping during COVID-19. This methodological innovation is congruent with asset-based community development (ABCD) to minimize arbitrariness in sampling decisions and advance health equity in our cities.

